# Editorial: Pre-natal and post-natal environmental impacts on metabolic control

**DOI:** 10.3389/fendo.2025.1583915

**Published:** 2025-03-18

**Authors:** David J. Hill, Daniel B. Hardy, Luis Sobrevia, Gernot Desoye

**Affiliations:** ^1^ Lawson Research Institute, St. Joseph’s Health Care, London, ON, Canada; ^2^ Department of Physiology and Pharmacology, Schulich School of Medicine and Dentistry, Western University, London, ON, Canada; ^3^ Cellular and Molecular Physiology Laboratory (CMPL), Department of Obstetrics, Division of Obstetrics and Gynaecology, School of Medicine, Faculty of Medicine, Pontificia Universidad Católica de Chile, Santiago, Chile; ^4^ Medical School (Faculty of Medicine), São Paulo State University (UNESP), Sao Paulo, Brazil; ^5^ Department of Physiology, Faculty of Pharmacy, Universidad de Sevilla, Sevilla, Spain; ^6^ University of Queensland Centre for Clinical Research (UQCCR), Faculty of Medicine and Biomedical Sciences, University of Queensland, Brisbane, QLD, Australia; ^7^ Gernot Desoye, Medical University of Graz, Graz, Austria

**Keywords:** fetus, pregnancy, metabolism, nutrition, type 1 diabetes, vitamin D, ERAS

Diverse environmental and maternal behavioral factors influence the future metabolic health of the offspring, including the pre-pregnancy health of the mother, the *in utero* environment, and early childhood development. Environmental risk factors are diverse and include maternal stress, nutrition, environmental pollutants, and lifestyle choices such as recreational drugs and alcohol. Such variables can impact diverse fetal tissues including the pancreas, liver, kidneys, skeletal muscle, adipose tissue, cardiovascular system, and brain. The placenta is a key mediator between maternal metabolic stress and altered fetal growth and development. Substantial gaps exist in our understanding of how individual organs and tissues are modified in both form and function by early life stressors, how limitations in tissue plasticity combine to build risk for adult disease, and where potential opportunities exist for metabolic rescue before or after birth.

The intent of this Research Topic was to illustrate key structural and functional developmental changes to organs and tissues that arise in response to sub-optimal environmental conditions during pregnancy and neonatal life, and how these can mutually build risk for adult metabolic diseases. Mechanistic changes include altered tissue stem cell populations, altered hormone and growth factor presence, epigenetic modifications in key genes regulating metabolism, and cellular stress. Recreational drugs such as cannabinoids present an unknown paradigm of risk to the offspring in addition to other maternal lifestyle choices. Understanding the extent of environmental impacts on tissue plasticity and the potential for when and how these might be reversed is essential to preventing future metabolic diseases.

The review by Hill and Hill focuses on the implications of maternal under- or overnutrition during pregnancy as key factors that alter fetal development, birth size, and the future metabolic health early trajectory of the child. Pregnancy involves adaptive changes to metabolism in the mother as a result of placental endocrinology. Maternal glycemic control and fetal organ development are compromised by either under- or overnutrition. In the fetus this includes long-lasting changes to pancreatic beta-cell mass and glucose-stimulated insulin release, adiposity, and insulin resistance. The cellular and molecular mechanisms identified include premature aging of fetal tissues, epigenetic changes to key genes regulating tissue metabolic function, altered progenitor cell number, developmental changes to the central nervous system, and an altered set point for basal circulating glucocorticoid levels. Importantly, the plasticity of metabolically-active tissues to accommodate metabolic stress in the offspring is compromised. Lifestyle and therapeutic interventions in the mother can improve her metabolic control and reduce the risk of abnormal birth size, but their effectiveness in changing the adverse metabolic trajectory in the offspring appear limited so far.

The paper Knuth et al. focuses on how a deficiency in vitamin D in early life might alter future hepatic cholesterol metabolism. A deficiency in vitamin D *in utero* was shown previously to result in metabolic dysfunction-associated steatotic liver disease (MASLD) in rat offspring. In the present study genetically inbred mouse strains were used where pregnant dams were maintained on a vitamin D deficient or sufficient diet throughout pregnancy and until weaning. The male offspring were thereafter fed vitamin-D sufficient diet and examined at 8-9 weeks of age. Profound changes were found in the liver transcriptome of mice deficient in vitamin D in early life with altered gene transcription associated with tissue development, inflammation, cholesterol biosynthesis, and energy metabolism. Transcriptional differences were accompanied by a general DNA hypomethylation suggesting long-lasting epigenetic changes in transcriptional control. Importantly, these changes could not be reversed by vitamin D supplementation after weaning, demonstrating the importance of vitamin D in early life in determining the trajectory of liver health.

The risk of developing Type 1 diabetes mellitus (T1DM) has been associated previously with an adverse intra-uterine environment and the paper by Fernandez Trigo et al. extends these studies to the importance of the intestinal microbiome using the non-obese diabetic (NOD) mouse model. Animals were bred in germ-free conditions and offspring colonized with a microbiome by exposure to a non-obese female mouse commencing either at birth or at weaning. Mice colonized at birth showed a subsequent reduction in the development of T1DM compared to those colonized at weaning with a different profile of intestinal regulatory T cells. The paper highlights the importance of the microbiome during early postnatal life in determining an autoimmune T cell signature, and the risk of T1DM.

Lastly, Zhou et al. examined how the clinical management at delivery of women with gestational diabetes mellitus (GDM) can alter maternal and newborn health. GDM is often associated with large-for-gestational age birth size and an increased risk of adult chronic diseases in the offspring and developing Type 2 diabetes mellitus in the mother. A retrospective cohort study compared perioperative management options during caesarean section. An ‘Enhanced recovery after surgery’ protocol (ERAS) was compared to a normally managed group of women with GDM. ERAS includes a pre-operative low carbohydrate drink which induces anabolism through an increase in insulin release. This can stabilize perioperative glycemia and prevent post-operative complications. However, the impact on metabolism in women with GDM was unclear. Results showed that the differences in preoperative glucose levels between ERAS and control group were minimal, but the ERAS group experienced a substantial decrease in composite adverse outcome, including perioperative maternal and neonatal hypoglycemia and hypertension. This could be anticipated to result in a lower referral to neonatal intensive care with reduced associated costs.

The papers included in this Research Topic highlight the range of environmental stressors during pregnancy that can impact the development of the fetus and the health of the offspring, from macronutrition to micronutrients and the host microbiome. The importance of early intervention and prevention strategies aimed at optimizing maternal health and the *in utero* environment is emphasized. While lifestyle and therapeutic interventions can improve maternal metabolic control and reduce the risk of abnormal neonatal outcomes, reversing the adverse metabolic programming remains a major challenge. Further, care protocols surrounding birth can also help to ensure neonatal health.

